# Proteomic analysis reveals the aging-related pathways contribute to pulmonary fibrogenesis

**DOI:** 10.18632/aging.205355

**Published:** 2023-12-22

**Authors:** Tingwei Zhang, Xinglong Yuan, Mengqi Jiang, Bo Liu, Nailiang Zhai, Qian Zhang, Xiaodong Song, Changjun Lv, Jinjin Zhang, Hongbo Li

**Affiliations:** 1 Department of Respiratory and Critical Care Medicine, Binzhou Medical University Hospital, Binzhou Medical University, Binzhou 256603, China; 2 Department of Cellular and Genetic Medicine, Binzhou Medical University, Yantai 264003, China; 3 Department of Pathology, Binzhou Medical University Hospital, Binzhou Medical University, Binzhou 256603, China

**Keywords:** aging, pulmonary fibrosis, proteomics, mechanical force, ferroptosis, autophagy, mitochondrial, senescence-inducing

## Abstract

Aging usually causes lung-function decline and susceptibility to chronic lung diseases, such as pulmonary fibrosis. However, how aging affects the lung-fibrosis pathways and leads to the occurrence of pulmonary fibrosis is not completely understood. Here, mass spectrometry-based proteomics was used to chart the lung proteome of young and old mice. Micro computed tomography imaging, RNA immunoprecipitation, dual-fluorescence mRFP-GFP-LC3 adenovirus monitoring, transmission electron microscopy, and other experiments were performed to explore the screened differentially expressed proteins related to abnormal ferroptosis, autophagy, mitochondria, and mechanical force *in vivo*, *in vitro*, and in healthy people. Combined with our previous studies on pulmonary fibrosis, we further demonstrated that these biological processes and underlying molecular players were also involved in the aging process. Our work depicted a comprehensive cellular and molecular atlas of the aging lung and attempted to explain why aging is a risk factor for pulmonary fibrosis and the role that aging plays in the progression of pulmonary fibrosis. The abnormalities of aging triggered an increase in mechanical force and ferroptosis, autophagy blockade, and mitochondrial dysfunction, which often appear during pulmonary fibrogenesis. We hope that the elucidation of these anomalies will help to enhance our understanding of senescence-inducing pulmonary fibrosis, thereby guiding the use of anti-senescence as an entry point for early intervention in pulmonary fibrosis and age-related diseases.

## INTRODUCTION

Aging is a complex physiological and pathological process characterized by many hallmarks, such as genomic instability, epigenetic alteration, loss of proteostasis, mitochondrial dysfunction, telomere attrition, and abnormal autophagy [1, 2]. Currently, the population is aging at a rate never seen before in human history. The number of adults aged over 65 globally is expected to increase from 617 million today to over 2 billion by 2050 and will account for 20% of the world’s population [3], suggesting that the population is aging at a rate never before seen in human history. To meet the important challenge of global health as the number of elderly adults grows, a pressing need exists to expand our understanding of the underpinnings of aging biology with the age-related diseases [4]. During aging, the physiological integrity is gradually lost, leading to impaired function of organs and increased vulnerability to death. It is the largest risk factor for a multitude of age-related diseases including pulmonary fibrosis, arteriosclerosis, Alzheimer’s disease, cancer, cardiovascular disease, type 2 diabetes, and osteoarthritis [5, 6]. These age-related diseases seriously affect people’s physical health and quality of life and pose certain challenges to healthcare and socioeconomics [7]. However, how aging affects the different functions of various senescent organs and leads to the occurrence of different diseases is not completely understood.

Aging can exacerbate lung function decline and susceptibility to chronic lung disease. Pulmonary fibrosis is a chronic and fatal lung disease that has so far been poorly treated with antifibrosis, immunosuppressive, and other complementary therapies [8, 9]. The incidence and severity of pulmonary fibrosis increases with age, suggesting that age is a major risk factor for IPF [10]. Aging can aggravate lung-function decline and susceptibility to chronic lung diseases. An atlas of the aging lung mapped by single-cell transcriptomics and deep-tissue proteomics uncovers extracellular-matrix remodeling in old mice [11], which is also the main clinical manifestation of pulmonary fibrosis. However, the mechanisms underlying the susceptibility to pulmonary fibrosis associated with aging are unknown.

Our previous studies on pulmonary fibrosis confirmed that abnormal biological processes such as autophagy blockade, abnormal ferroptosis, and mitochondrial dysfunction promote pulmonary fibrogenesis [12–15], which lays the foundation for the study of aging and pulmonary fibrosis in this paper. In the current work, we used mass spectrometry-based proteomics to chart the lung proteome of young and old mice. Based on the screened differentially expressed proteins related to abnormal ferroptosis, autophagy, mitochondrial dysfunction, and mechanical force, and combined with our research on pulmonary fibrosis, we demonstrated that these biological processes and the underlying molecular players were involved in aging and pulmonary fibrosis. We further attempted to explain why aging was a risk factor for pulmonary fibrosis. These abnormalities of aging triggered an increase in mechanical force and ferroptosis, autophagy blockade, and mitochondrial dysfunction, which often appear during pulmonary fibrogenesis. We hope that their elucidation can improve our understanding of senescence-inducing pulmonary fibrosis and thus guide the search for potential therapeutic targets of earlier interventions for pulmonary fibrosis and age-related diseases.

## RESULTS

### Overview of aging impact on the mouse lung

**Figure 1:**
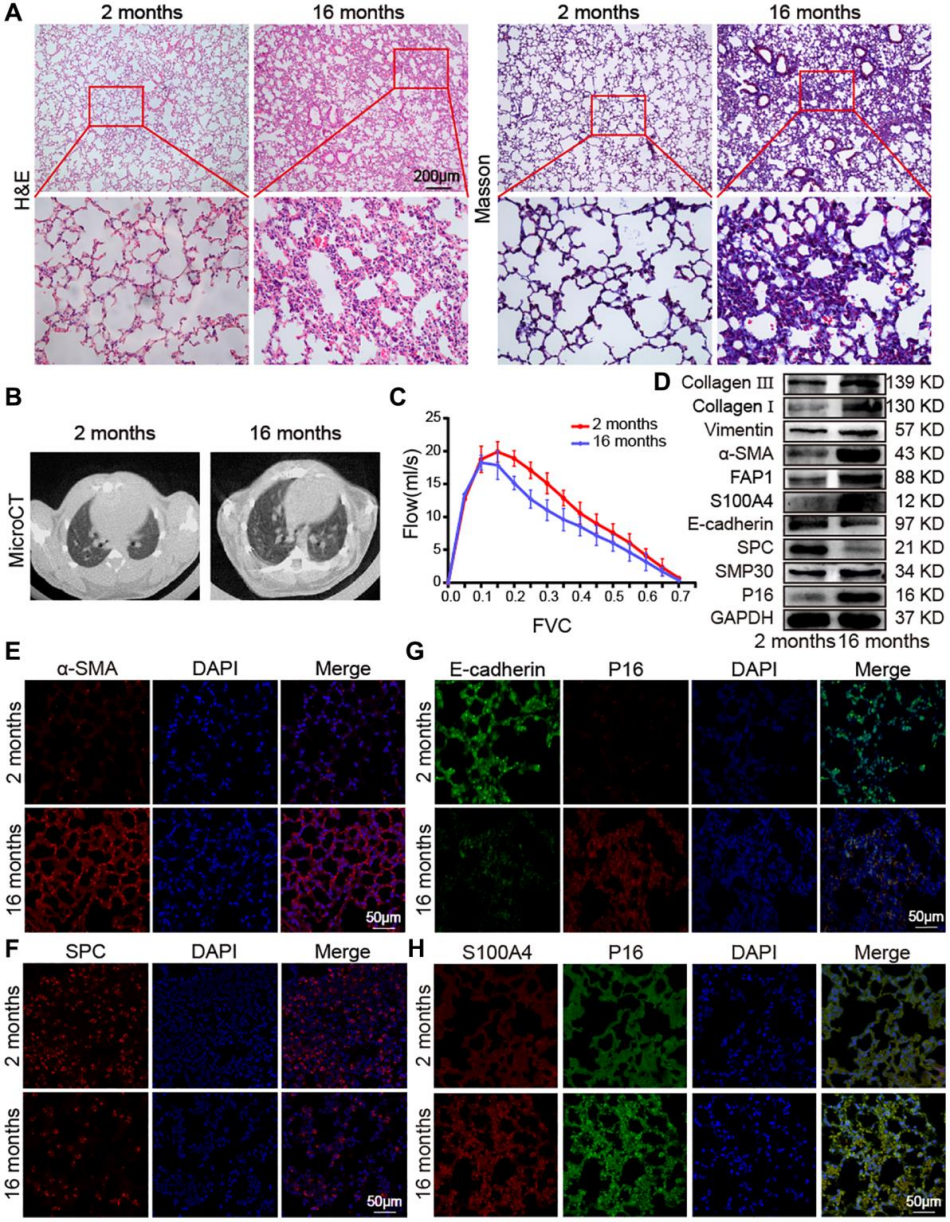
**Impact of aging on mouse lung.** (**A**) H&E and Masson staining showed that the alveolar structure of the lung tissue of 2-month-old mice was clear and intact, whereas the lung tissue of 16-month-old mice had more collagen deposition, damaged alveolar structure, and thickened alveolar walls. (**B**) MicroCT imaging for small animal demonstrated that some old mice had fibrous strip shadows and thickened interlobular septa compared with young mice. (**C**) FVC results showed that pulmonary function decreased in aging mice. (**D**) Western blot showed that collagen III, collagen I, vimentin, α-SMA, FAP1, S100A4, SMP30, and P16 increased significantly in aging mice, but E-cadherin and SPC decreased. (**E**) Immunofluorescence staining showed that α-SMA expression in lung tissues of aging mice was higher than in those of young mice. (**F**) Immunofluorescence images showed that SPC expression was significantly lower in lung tissue of 16-month-old mice than in that of 2-month-old mice. (**G**) Double immunofluorescence staining showed that P16 increased in aging mice and decreased in young mice. E-cadherin decreased in aging mice and increased in young mice. (**H**) Double immunofluorescence staining showed that the expression of S100A4 and P16 in the lung tissue of aging mice were significantly higher than in that of young mice.

**Figure 2:**
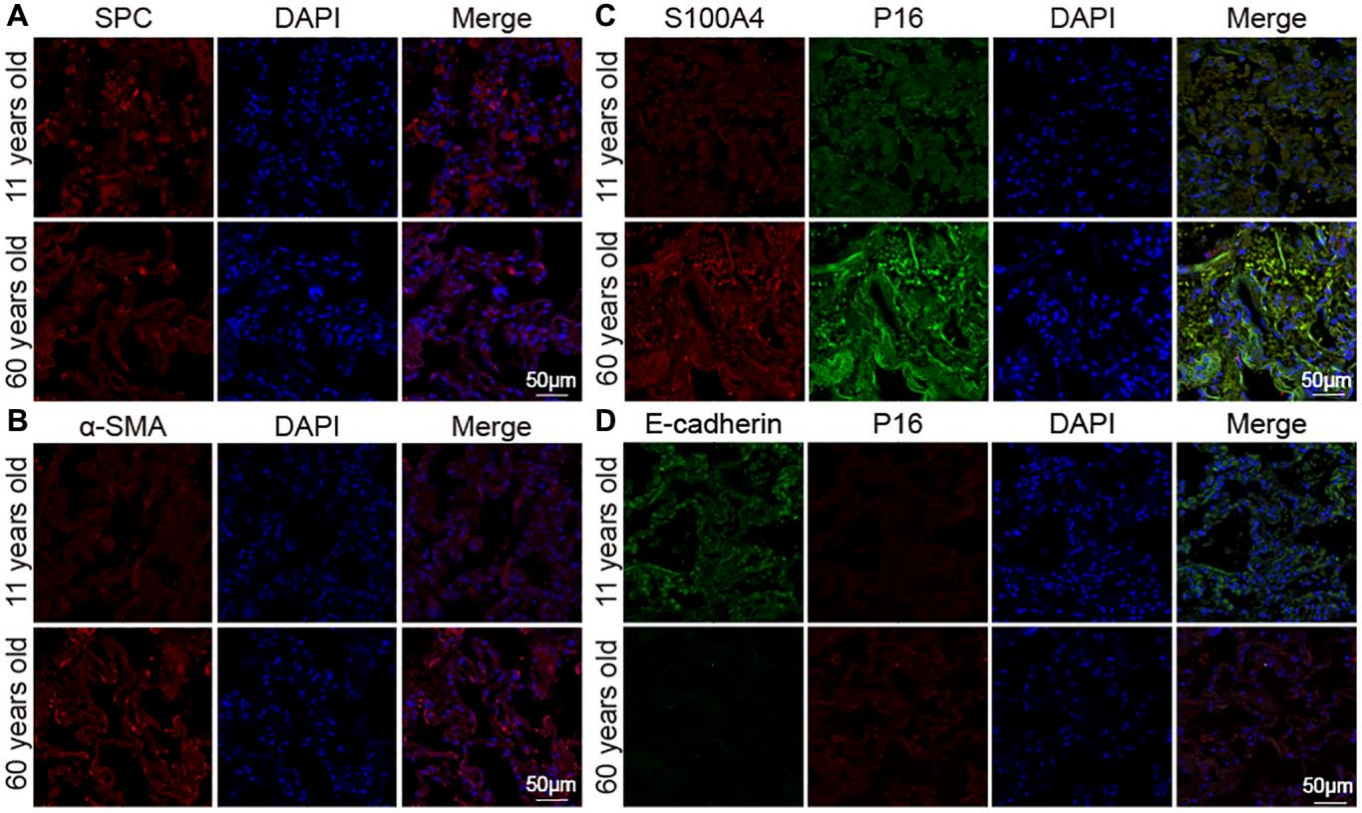
**Expression of marker proteins in healthy people.** (**A**) Immunofluorescence images showed decreased SPC in lung tissue of the elderly. (**B**) Immunofluorescence images showed increased α-SMA expression in aging human lung tissue than in young. (**C**) Double immunofluorescence staining showed that the expression of S100A4 and P16 were significantly higher in aged human lung tissues than in young lung tissues. (**D**) Double immunofluorescence staining showed that P16 increased in the old and decreased in the young. E-cadherin decreased in the old and increased in the young.

Aging impact on mouse lung was analyzed between 16-month-aging mice and 2-month-old mice, hereafter denoted as the aging and young groups, respectively. Hematoxylin–eosin (H&E) and Masson staining depicted the senescence morphological alterations in aging mouse lungs. Images presented that the lung-tissue structure was clear and that bronchial, alveolar, and alveolar septa had a normal organizational structure. The blood–air barriers were also distinct and intact in the young group. In the aging group, the alveolar wall thickened, the alveolar cavity became smaller, the framework was disordered, and excess collagen was deposited compared with those in the young group (Figure 1A). Micro computed tomography (MicroCT) imaging for small animal demonstrated that some aging mice had obvious fibrous strip shadows and thickened interlobular septa compared with young mice (Figure 1B). The forced vital capacity (FVC) showed that pulmonary function decreased in aging mice (Figure 1C). Results of Western blot and immunofluorescence staining analyses illustrated that senescence-marker proteins such as P16 and senescence-marker protein 30 (SMP30), as well as fibrosis markers including collagen III, collagen I, vimentin, alpha-smooth muscle actin (α-SMA), fibroblast activation protein 1 (FAP1), and S100 calcium binding protein A4 (S100A4), increased. However, epithelial markers such as E-cadherin and surfactant protein C (SPC) decreased in aging mice compared with those young mice (Figure 1D–1F). Double immunofluorescence staining showed that P16 increased in aging mice and decreased in young mice, whereas E-cadherin expression decreased in aging mice and increased in young mice. This finding indicated that aging induced the loss of epithelial progenitor cell function and/or numbers (Figure 1G). Double immunofluorescence staining further demonstrated that the fibroblastic marker S100A4 and p16-INK4a (P16) increased in aging mice, indicating that fibroblasts increased in aging (Figure 1H). Then, the expression of SPC, α-SMA, E-cadherin, S100A4, and P16 were confirmed in young and aging human lung tissues by laser scanning confocal microscopy. Immunofluorescent images showed that the expression of the alveolar epithelial cell marker SPC was lower in aging human lung tissue than in the young group (Figure 2A). However, α-SMA expression was higher in aging human lung tissue than in the young group, indicating that aging was accompanied by fibrotic changes (Figure 2B). Double immunofluorescence staining demonstrated that S100A4 and P16 increased in aging human lung tissues and decreased in young tissues, whereas E-cadherin expression decreased in aging tissues and increased in young ones (Figure 2C, 2D). These results were consistent with those in mice. These findings suggested that aging can induce an increase in senescence-marker and fibrosis-marker proteins.

### Proteomic analysis in aging mice

**Figure 3:**
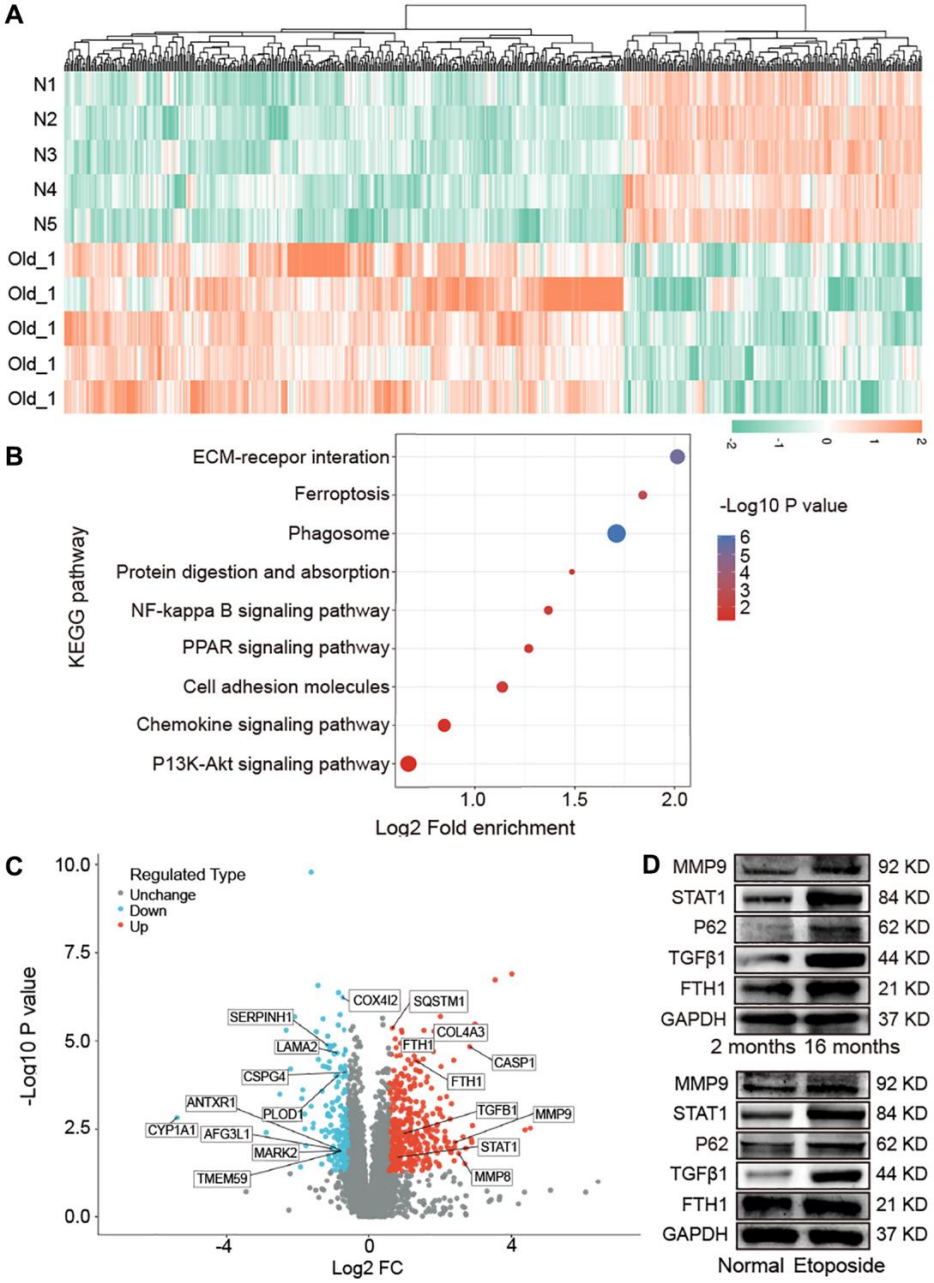
**Impact of aging on mouse lung proteomics.** (**A**) Hierarchical clustering of protein analysis. Blue to red indicate low-to-high expression levels. (**B**) KEGG pathway analysis exhibited that the screened differentially expressed proteins were enriched in pathways including ECM−receptor interaction, ferroptosis, phagosome, protein digestion and absorption, NF-kappa B signaling pathway, PPAR signaling pathway, and cell adhesion molecules. (**C**) Differentially expressed proteins related to ferroptosis, autophagy, mitochondria, and fibrosis were screened. (**D**) Western blot assay identified that the expression levels of FTH1, TGFβ1, P62, STAT1, MMP9 increased in aging mice compared with those in young mice. The *in vitro* result was consistent with the *in vivo* one.

Proteomics analysis using liquid chromatography with tandem mass spectrometry was performed to analyze the impact of aging on lung proteomics between young and mice. A total of 579 differentially expressed proteins, with 378 upregulated and 201 downregulated ones, were found in aging mice compared with the young control (Figure 3A). The Kyoto Encyclopedia of Genes and Genomes (KEGG) pathway was used to analyze the screened differentially expressed proteins. Results exhibited that these proteins were enriched in the pathways of extracellular matrix (ECM)−receptor interaction, ferroptosis, phagosome, protein digestion and absorption, nuclear factor kappa-B (NF-kappa B) signaling pathway, peroxisome proliferator-activated receptor (PPAR) signaling pathway, and cell adhesion molecules (Figure 3B). Part of the differentially expressed proteins information was listed in Table 1. According to *P* < 0.05 and log2 fold change >2, we further screened ferroptosis-associated differentially expressed proteins (FTH1, STAT1, SQSTM1/P62, TGFβ1, MMP9, MMP8, and FTL1), autophagy-associated differentially expressed proteins (SQSTM1/P62, MMP9, MMP8, and PLOD1), mitochondrion-associated differentially expressed proteins (MMP9, MMP8, and AFG3L1), and fibrosis-associated differentially expressed proteins (TGFβ1, MMP9, and COL4A3). Among them, the upregulated proteins included FTH1, STAT1, SQSTM1/P62, TGFβ1, MMP9, MMP8, COL4A3, FTL1, and CASP1, and the downregulated ones included CYP1A1, COX4I2, AFG3L1, SERPINH1, LAMA2, CSPG4, PLOD1, TMEM59, MARK2, and ANTXR1 (Figure 3C). Then, we randomly selected five proteins to confirm the proteomics analysis. Western blot manifested that MMP9, STAT1, FTH1, P62, and TGFβ1 increased in aging mice than those in young mice. Etoposide was used to establish an aging cell model in a human fetal lung fibroblast medical research council cell strain-5 (MRC-5) cell line. The expression of MMP9, STAT1, FTH1, P62, and TGFβ1 increased in the etoposide group compared with the control group (Figure 3D). The *in vivo* and *in vitro* results were consistent with the mass spectra.

**Table 1:** Part of the differentially expressed proteins information.

**Protein description**	**Protein abbreviation**	**Function**	**Up/Down**	**Old/*N* ratio**
Caspase-1	CASP1	Regulate mitochondrial membrane potential	Up	7.10
Matrix metallopeptidase 8	MMP8	Regulate extracellular matrix; regulate the autophagy process	Up	5.73
Matrix metallopeptidase 9	MMP9	Promote fibroblast proliferation; associated with mitochondria, oxidative stress, mechanical forces and iron ions	Up	5.33
Collagen alpha-3(IV) chain	COL4A3	Regulate cell proliferation; activate collagen signaling pathway	Up	3.56
Integrin alpha-IIb	ITGA2B	Promote cell adhesion; promote extracellular matrix deposition	Up	3.02
Collagen alpha-1(IV) chain	COL4A1	Activate collagen signaling pathway	Up	2.93
Integrin alpha-L	ITGAL	Organize extracellular matrix	Up	2.73
Ferritin light chain 1	FTL1	Regulate the sequestering of iron ion	Up	2.47
Ferritin heavy chain 1	FTH1	Regulate iron ion; regulate cell proliferation; regulate fibroblast proliferation	Up	2.41
Transforming growth factor beta 1	TGFβ1	Regulate mitotic cell cycle; response to mechanical stimulus; promote collagen production and tissue remodeling; regulate fibroblast proliferation	Up	2.00
Collagen Type VI Alpha 2 Chain	COL6A2	Promote extracellular matrix deposition	Up	1.88
Signal transducer and activator of transcription 1	STAT1	Response to mechanical stimulus; regulate smooth muscle cell proliferation;	Up	1.77
Apolipoprotein A1	APOA1	Promote cell-substrate adhesion; promote actin filament bundle assembly; organize cytoskeleton	Up	1.72
Sequestosome-1 /P62	SQSTM1/P62	Regulate mitochondrial metabolism; regulate autophagy process	Up	1.58
Chondroitin sulfate proteoglycan 4	CSPG4	Response to stress	Down	0.64
Heterogeneous nuclear ribonucleoprotein A1	HNRNPA1	regulate fibroblast growth factor receptor signaling pathway	Down	0.63
Janus kinase 2	JAK2	actin polymerization or depolymerization; regulate smooth muscle cell proliferation	Down	0.62
Cytochrome c oxidase subunit 4 isoform 2	COX4I2	regulate mitochondrial electron transport	Down	0.59
Transmembrane protein 59	TMEM59	regulate autophagy process; regulate protein glycosylation in Golgi	Down	0.57
Anthrax toxin receptor 1	ANTXR1	organize actin cytoskeleton; regulate extracellular matrix	Down	0.57
Microtubule affinity-regulating kinase 2	MARK2	regulate mitochondrion autophagy; organize cytoskeleton	Down	0.56
Procollagen-lysine, 2-oxoglutarate 5-dioxygenase 1	PLOD1	response to stress; regulate oxidation-reduction process	Down	0.54
Laminin subunit alpha-2	LAMA2	involved in cellular development processes	Down	0.53
AFG3-like protein 1	AFG3L1	regulate mitochondrial metabolism	Down	0.48
Serpin Family H Member 1	SERPINH1	regulate collagen fibril	Down	0.44
Cytochrome P450 1A1	CYP1A1	regulate mitotic cell cycle; regulate iron ion; involved in reactive oxygen species metabolism	Down	0.02

**Figure 4:**
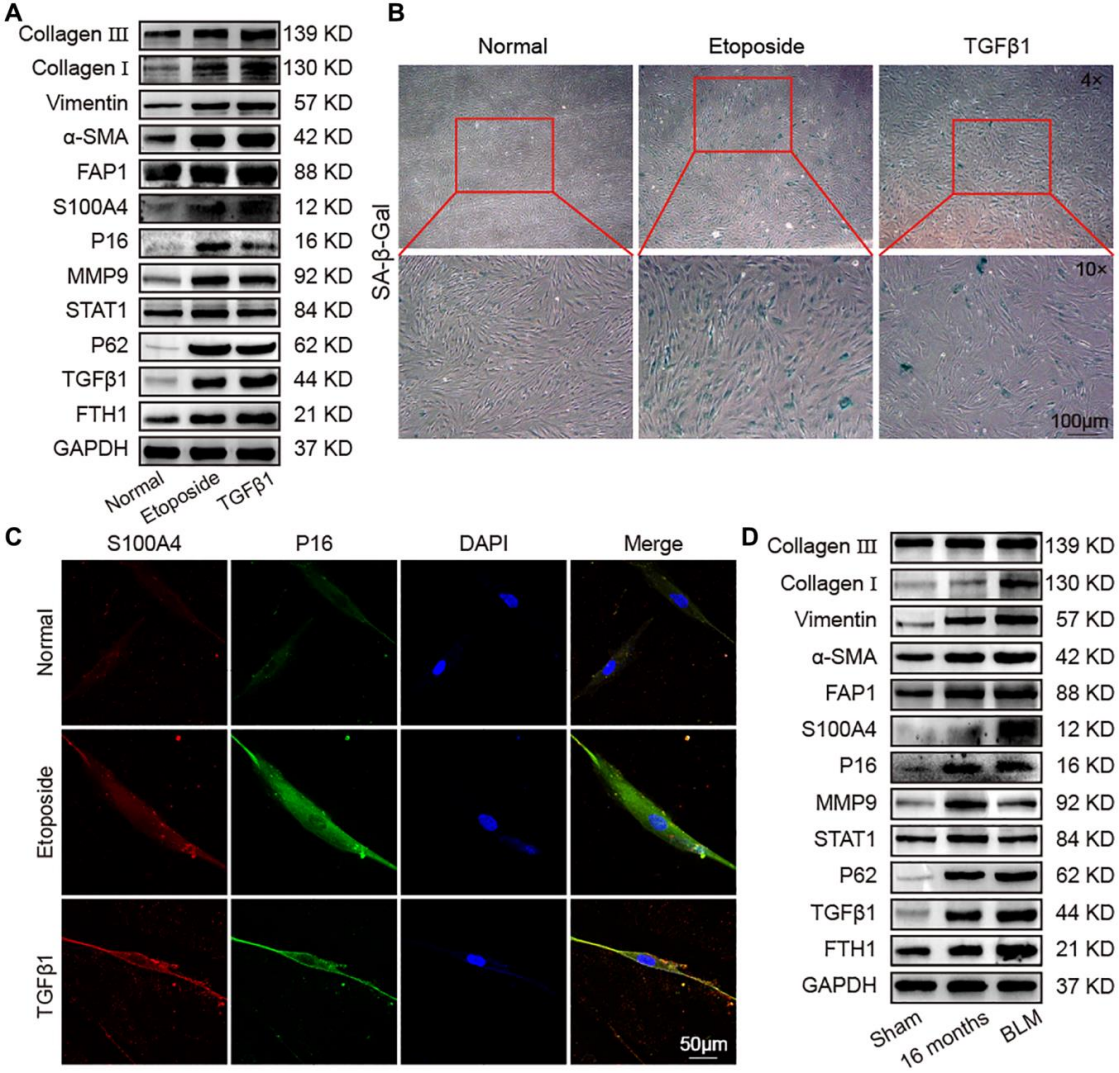
**Identification of senescence and fibrosis markers in an aging cell model, fibrotic cell model, and fibrotic mouse model.** Aging cell models were established in MRC-5 cells using 10 μM etoposide, and 5 ng/mL TGFβ1 was used to establish a fibrotic cell model in MRC-5 cell. The BLM model group was sprayed with 5 mg/kg BLM through the trachea by using a Penn–Century MicroSprayer. (**A**) The expression levels of collagen III, collagen I, vimentin, α-SMA, FAP1, S100A4, P16, MMP9, STAT1, P62, TGFβ1, and FTH1 increased in the etoposide and TGFβ1 groups compared with those in normal mice. (**B**) SA-β-gal-positive cells increased in the TGFβ1-stimulated group, but the etoposide group had more β-gal-positive cells than the normal and TGFβ1 groups. SA-β-gal-positive cells are blue. (**C**) Double immunofluorescence staining showed that S100A4 and P16 were significantly highly expressed in the etoposide and TGFβ1 groups, but S100A4 in the TGFβ1 group was higher than that in the etoposide group, and P16 in the etoposide group was higher than that in the TGFβ1 group. (**D**) The expression levels of collagen III, collagen I, vimentin, α-SMA, FAP1, S100A4, P16, MMP9, STAT1, P62, TGFβ1, and FTH1 in lung tissue of aged and fibrotic mice were significantly higher than that of the sham group.

Lung proteomic analysis of aging mice revealed that many fibrosis proteins were higher than young mice, consistent with the idea that aging was a major risk factor for pulmonary fibrosis. To clarify the association between aging and pulmonary fibrosis, TGFβ1 was used to establish a fibrotic cell model in MRC-5 cells. Western blot confirmed that collagen III, collagen I, vimentin, α-SMA, FAP1, S100A4, P16, MMP9, STAT1, FTH1, P62, and TGFβ1 increased in pulmonary fibrosis in an *in vitro* model compared with the control group. The expression of the fibrosis-marker proteins collagen III, collagen I, vimentin, α-SMA, FAP1, and S100A4 in the fibrotic group were significantly higher than that in the etoposide group, but the expression of the senescence-marker protein P16 was significantly lower than that in the etoposide group (Figure 4A). Senescence-associated β-galactosidase (SA-β-gal) staining exhibited that the fibrotic group had more β-gal-positive cells compared with the normal group but had less than the aging group (Figure 4B). Fluorescence images showed that the expression of S100A4 and P16 in the aging and fibrotic groups were significantly higher than that in the normal group, but the fibroblastic marker S100A4 in the fibrotic group was higher than that in the aging group, and the senescence-marker protein P16 in the aging group was higher than that in the fibrotic group (Figure 4C). To further prove these results, we used bleomycin (BLM)-treated mice as an animal fibrosis model. Western blot showed that the expression trends of collagen III, collagen I, vimentin, α-SMA, FAP1, S100A4, P16, MMP9, STAT1, P62, TGFβ1, and FTH1 were consistent with those in the *in vitro* model (Figure 4D). These data manifested that fibrosis proteins increased during aging, and the senescence proteins also increased during fibrogenesis.

### Abnormal expression of key molecular players related to ferroptosis and autophagy in aging

**Figure 5:**
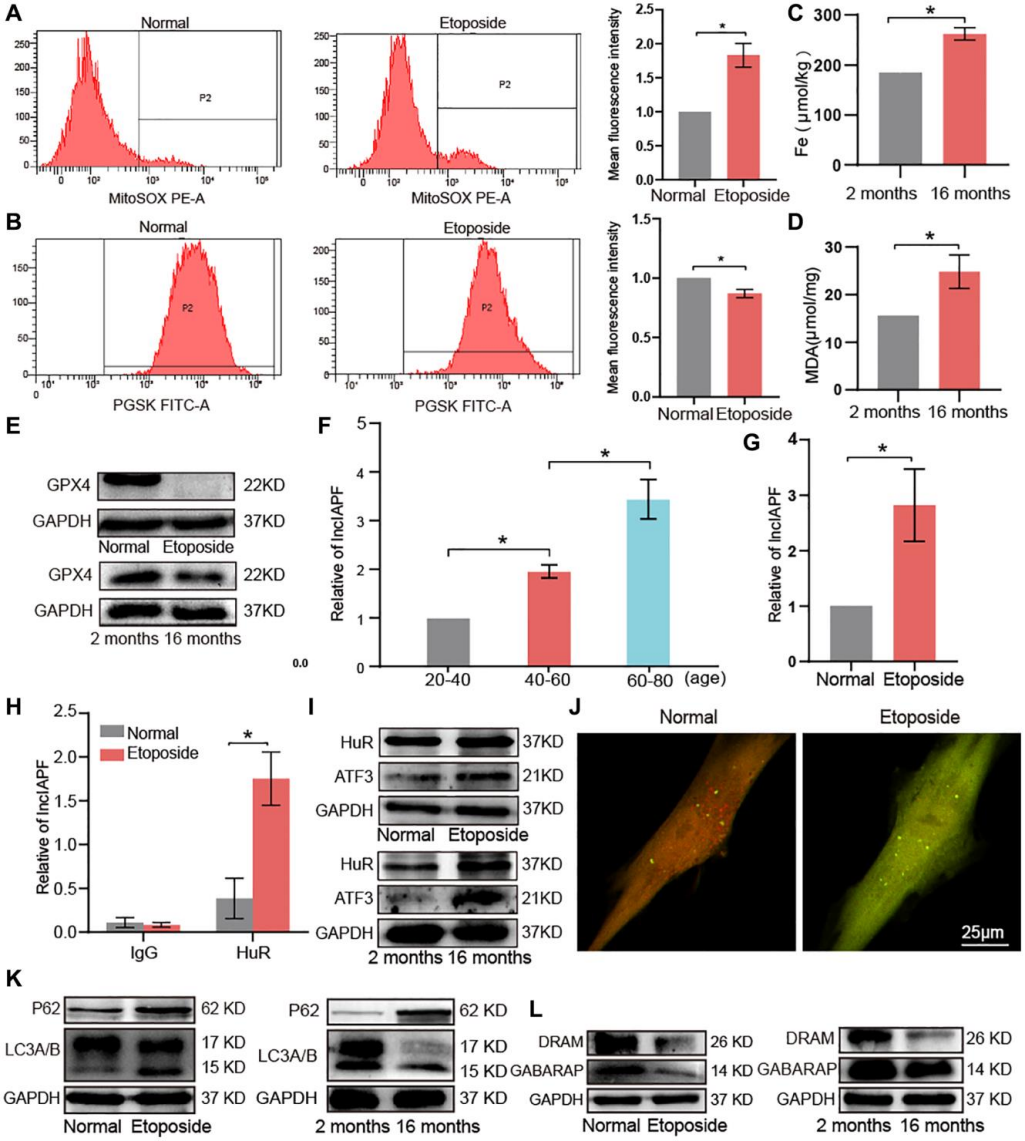
**Key molecular players of ferroptosis and autophagy signaling pathways in aging.** (**A**) Flow cytometry assay showed that ROS expression increased in senescent cells compared with normal ones. (**B**) Senescent cells expressed more ferric ions than normal ones. (**C**) Total iron increased in lung tissue of 16-month-old mice compared with the 2-month-old group. (**D**) MDA increased in lung tissues of 16-month-old mice compared with the 2-month-old group. (**E**) Western blot showed that GPX4 expression decreased in aging cells compared with normal cells *in vivo* and *in vitro*. (**F**) qRT-PCR results showed that lncIAPF expression increased with age in a group of healthy population. (**G**) qRT-PCR results showed that lncIAPF expression increased in the aging cell model than those in the normal one. (**H**) RIP results verified that HuR specifically concentrated large quantities of lncIAPF. (**I**) Western blot confirmed that the expression levels of ATF3 and HuR increased in the aging cell model and lung tissue compared with those in the normal group. (**J**) Application of dual-fluorescence mRFP-GFP-LC3 adenovirus monitoring to observe autophagy under laser confocal microscopy revealed that the number of yellow autophagosomes increased in the aging group compared with the normal one. (**K**) Western blot showed that P62 and LC3A/B increased in the aging group compared with the normal one. (**L**) Western blot showed that DRAM and GABARAP decreased in the aging group compared with the normal one. Each bar represents the mean ± SD; *n* = 3; ^*^p < 0.05.

Lung proteomic analysis demonstrated higher levels of FTH1, P62, and STAT1 in aging mice than those in the young group. In research about pulmonary fibrosis, FTH1 is involved in ferroptosis [13], whereas P62 and STAT1 are involved in autophagy [12]. Accordingly, the abnormal expression of molecules related to ferroptosis and autophagy in aging were selected for exploration. Ferroptosis is characterized by increased reactive oxygen species (ROS) and lipid peroxidation and decreased glutathione peroxidase 4 (GPX4) activity. Changes in ROS in the aging cell model were detected by flow cytometry. ROS increased in the aging cell model compared with normal cells (Figure 5A). Flow cytometry assay of phen green SK probe showed that fluorescence intensity decreased in senescent cells, indicating elevated levels of ferric ions (Figure 5B). To further verify the changes in iron content, we performed total iron content measurements in lung tissues of young and aging mice, showing that the total iron content in lung tissues of aging mice was significantly higher than that of the young group (Figure 5C). Malondialdehyde (MDA) assay showed increased MDA was in the lung tissue of aging mice (Figure 5D). Western blot revealed that GPX4 significantly decreased in the aging cells and mice. These data suggested that ferroptosis occurred in aging (Figure 5E).

Our previous study has shown that activating transcription factor 3 (ATF3)-activated lncIAPF-HuR complex accelerates pulmonary fibrosis by blocking autophagy [12]. Accordingly, we wondered whether the lncIAPF-mediated signal axis was also involved in aging, so we tested lncIAPF expression in a healthy population of different ages. Quantitative real-time polymerase chain reaction (qRT-PCR) results showed that lncIAPF expression increased with age in the healthy population (Figure 5F). In the aging cell model, lncIAPF expression also increased compared with the normal one (Figure 5G). RNA immunoprecipitation (RIP) results revealed that Hu antigen R (HuR) was specifically enriched by lncIAPF in aging cell model compared with normal cells to prove their binding relationship (Figure 5H). Western blot showed that the expression levels of that HuR and ATF3 increased significantly in senescent cells and lung tissue compared with those in the normal model (Figure 5I). Dual-fluorescence mRFP-GFP-LC3 adenovirus monitoring technology was used to observe autophagy by using a laser confocal microscope. Red fluorescence indicated autophagosome in normal autophagy. Yellow fluorescence indicated autophagosome in abnormal autophagy. This result showed that the number of yellow autophagosomes increased in aging compared with the normal one (Figure 5J). Protein-related autophagy including light chain 3 A/B (LC3A/B), P62, damage-regulated autophagy modulator (DRAM), and GABA receptor-associated protein (GABARAP) were measured. Results showed that LC3A/B and P62 increased, but DRAM and GABARAP decreased (Figure 5K, 5L). DRAM and GABARAP primarily regulated the late stage of autophagy, and combined with our previous research about autophagy [12, 16, 17], we inferred that autophagy was blocked in the later stage during aging. In other words, autophagosomes can form in the early stage, but they cannot be degraded in the later stage. All these results demonstrated that lncIAPF-mediated autophagic signal axis was also involved in aging.

### Mitochondrial dysfunction in aging model *in vivo* and *in vitro*

**Figure 6:**
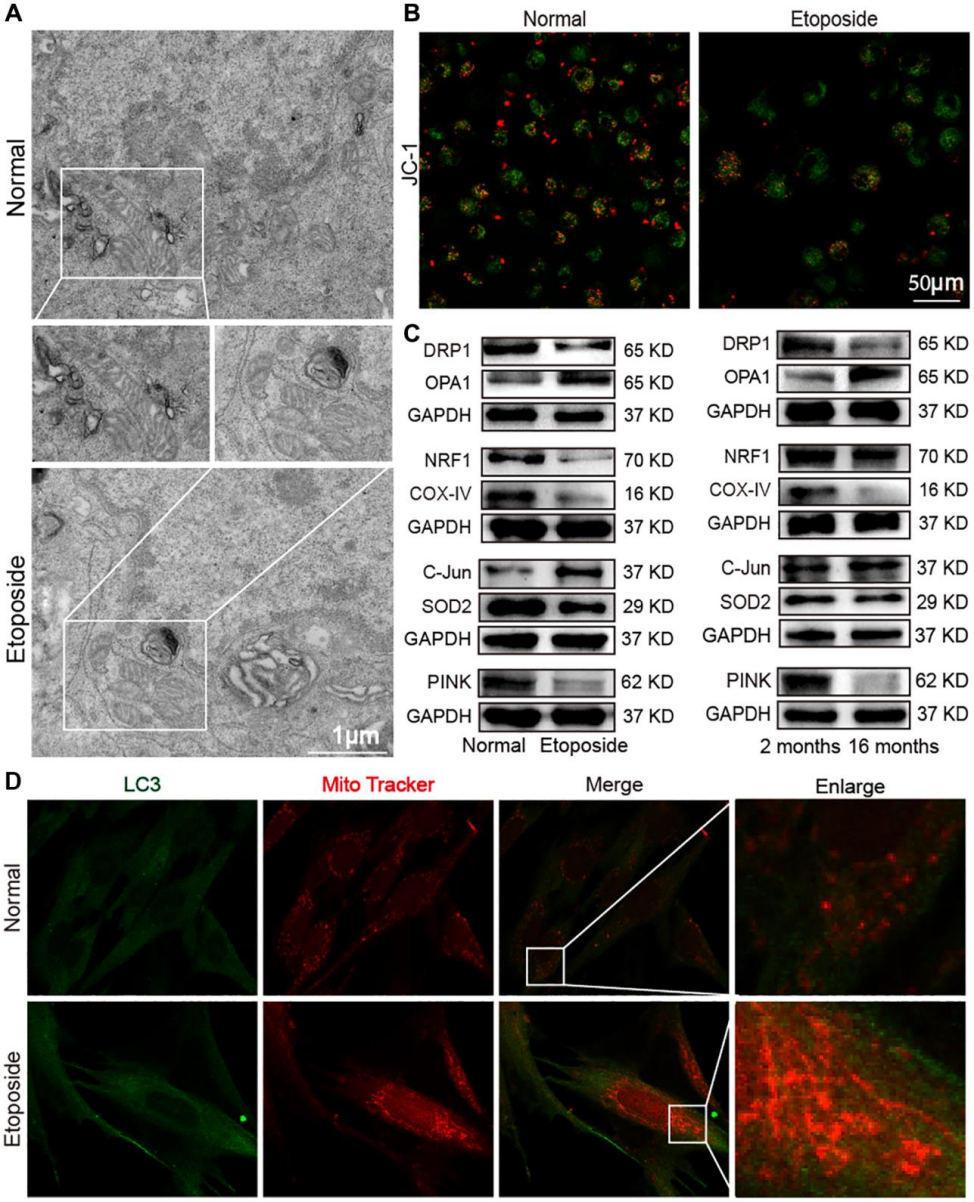
**Dysfunctional mitochondria in the aging model *in vivo* and *in vitro*.** (**A**) TEM examination showed mitochondrial crumpling, disrupted integrity of the outer mitochondrial membrane, and reduced mitochondrial cristae in the aging group. (**B**) Detection of mitochondrial membrane potential by laser scanning confocal microscopy revealed decreased red fluorescence and increased green fluorescence in senescent cells. Red fluorescence indicated JC-1 polymer; green fluorescence indicated JC-1 monomer. (**C**) Western blot showed increased expression levels of OPA1 and C-Jun in the senescent cell model and decreased expression levels of DRP1, NRF1, COX-IV, SOD2, and PINK with the same expression trend in the aging mice model. (**D**) Double immunofluorescence assay showed increased mitochondria (red fluorescence) and LC3 (green fluorescence) in senescent cells compared with normal ones. Mitochondria in the normal group showed an extended tubular structure, and in the etoposide group mitochondria were rounded and fragmented.

Mitochondria are involved in many biological processes including ferroptosis and autophagy, so mitochondrial dysfunction is an important component of different diseases associated with aging [18, 19]. Ferroptosis and autophagy are always accompanied by mitochondrial regulation, such as mitochondrial biogenesis, fusion/division, unfolded protein response processes, and mitophagy, so mitochondrial dysregulation in aging was studied. Morphological changes of mitochondria were first observed by the transmission electron microscopy (TEM). The images showed that in the etoposide group compared with the normal group, mitochondria were wrinkled, the integrity of the outer mitochondrial membrane was disrupted, and mitochondrial cristae were reduced (Figure 6A). Laser scanning confocal microscopy was used to examine the changes in mitochondrial membrane potential in the aging cell model. JC-1 forms polymers with red fluorescence in normal mitochondria. With decreased membrane potential, JC-1 became a monomer and showed green fluorescence. The change in ratio of red to green fluorescence served as an indicator of mitochondrial condition. This result showed that senescent cells had a lower mitochondrial membrane potential than normal cells (Figure 6B). Western blot detected that the mitochondrial fusion protein optic atrophy 1 (OPA1) expression increased, whereas mitochondrial division protein dynamin-related protein 1 (DRP1) expression decreased in the aging groups *in vivo* and *in vitro*. Similarly, mitochondrial production proteins such as nuclear respiratory factor 1 (NRF1) and cytochrome c oxidase IV (COX-IV) decreased in the aging groups, and the mitochondrial folded protein C-Jun increased and superoxide dismutase 2 (SOD2) decreased in the aging groups. Mitophagy-related protein phosphatase and tensin homolog-induced kinase (PINK) decreased in the aging groups compared with those in the young group (Figure 6C). MRC-5 cells were co-stained with Mitotracker Red CMXRos and anti-LC3 antibody. After Mito-Tracker Red CMXRos labeling of biologically active mitochondria, double immunofluorescence staining results showed that LC3 was highly expressed in the senescent group, with a significant increase in mitochondrial staining intensity, disruption of the mitochondrial three-dimensional meshwork, and rounded and fragmented mitochondrial appearance (Figure 6D). These results indicated that mitochondrial dysfunction occurred in aging model *in vivo* and *in vitro*.

### circANKRD42 mediated the aging-inducing mechanical forces

**Figure 7:**
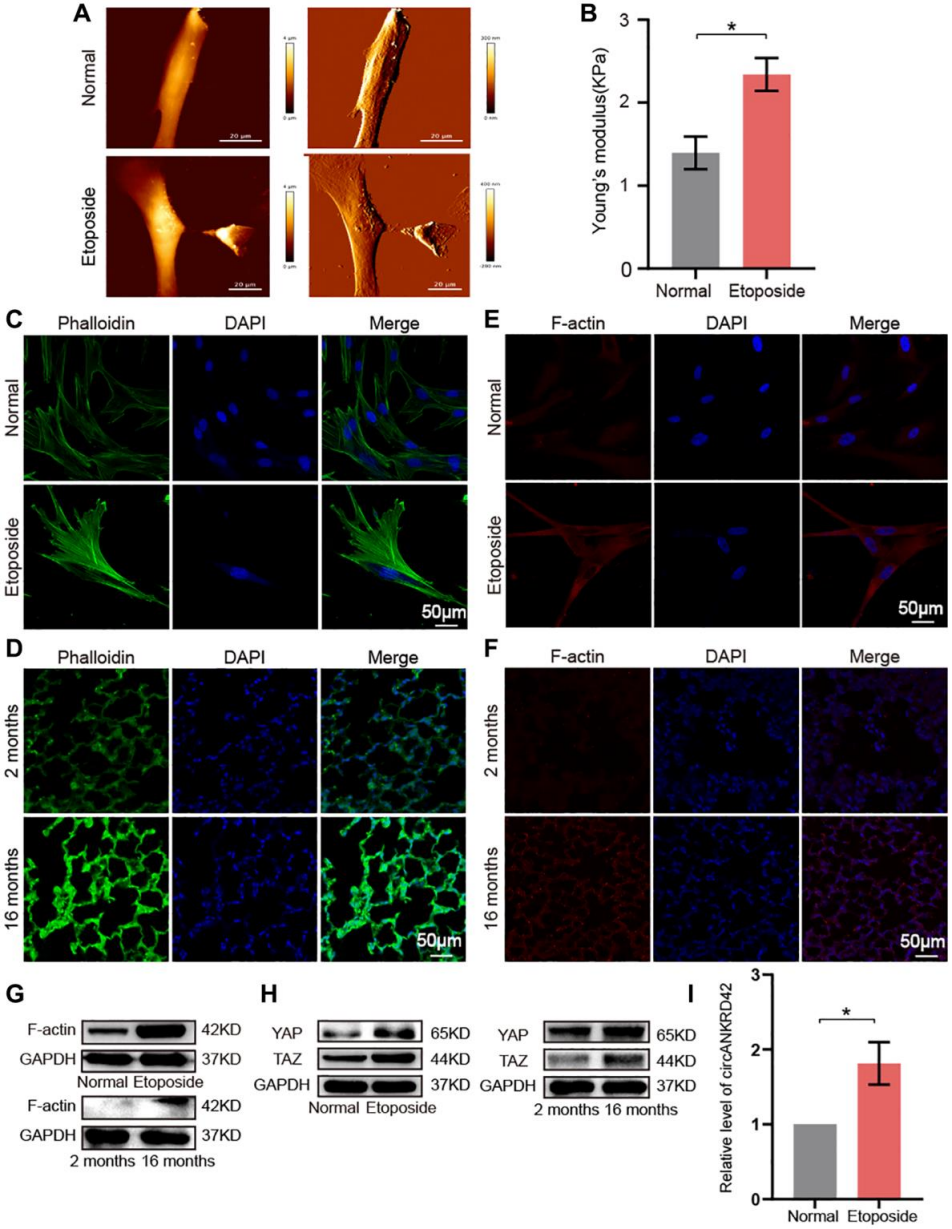
**circANKRD42 mediated the aging-inducing mechanical forces.** (**A**) AFM images showed that Young’s modulus increased in senescent cells compared with normal ones. (**B**) The reaction force of senescent cells was higher than that of normal cells. (**C**) Phalloidin staining depicted that the cytoskeleton became disordered and underwent disorganized expansion in the aging cell model compared with that in the normal one. (**D**) Phalloidin staining depicted that the cytoskeleton became disordered and underwent disorganized expansion in the lung tissue of 16-month-old mice compared with the 2-month-old ones. (**E**) Immunofluorescent staining showed that F-actin was highly expressed in aging cells compared with that in the normal one. (**F**) Immunofluorescent staining showed that F-actin was highly expressed in aging lung tissue than in the young. (**G**) Western blot showed that the expression level of F-actin increased in the aging cell and tissue model compared with those in the normal one. (**H**) Western blot showed that the expression levels of YAP and TAZ increased in the aging cell and tissue model compared with those in the normal one. (**I**) qRT-PCR result revealed that circANKRD42 expression increased in the aging cell model compared with those in the normal one. Each bar represents the mean ± SD; *n* = 3; ^*^p < 0.05.

**Figure 8:**
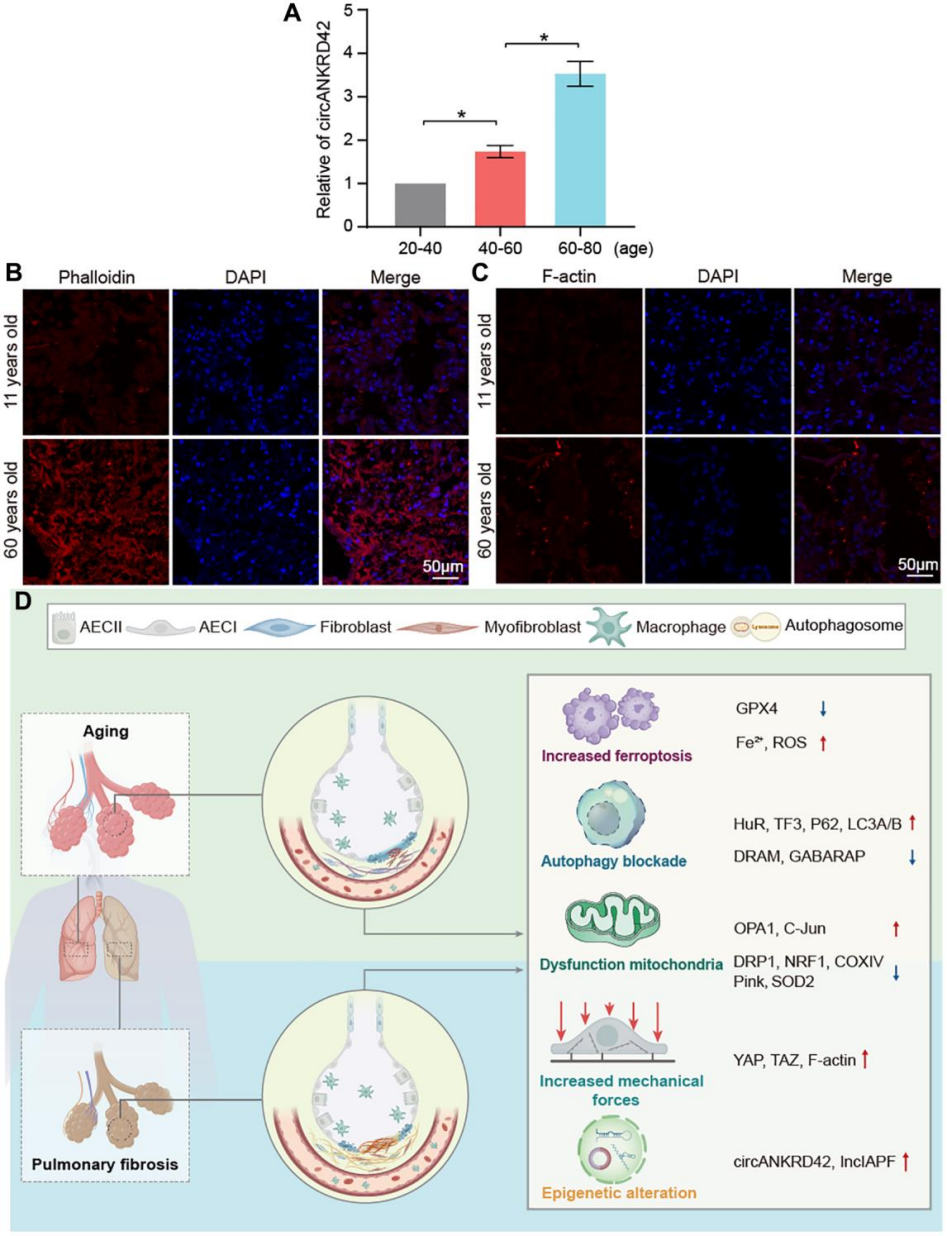
**Assessment of circANKRD42 and cytoskeleton in the healthy population and the key molecular players in aging and pulmonary fibrosis.** (**A**) qRT-PCR result showed that circANKRD42 expression increased with age in a healthy-population group. (**B**) Phalloidin staining depicted that the cytoskeleton became disorder and underwent disorganized expansion in aging humans compared with young ones. (**C**) Fluorescence images showed that F-actin increased in 60-year-old healthy people compared with that in 11-year-old ones. (**D**) Key molecular players were involved in aging and pulmonary fibrosis, including epigenetic alteration (lncIAPF and circANKRD42), mitochondrial dysfunction, abnormal ferroptosis, autophagy, and mechanical forces.

Given that vimentin, α-SMA, and collagen increase with aging, it inevitably leads to increased lung hardness and decreased lung elasticity. It is the most typical phenotype of the elderly lung and is also the most common phenotype of pulmonary fibrosis. circANKRD42 can mediate the crosstalk of mechanical stiffness and biochemical signal to promote lung fibrosis [14]. We further explored whether circANKRD42-mediated signal axis was also involved in aging. We took cell stiffness, cytoskeleton tension, and mechanical stress-related proteins as examples of the hardness and elasticity to measure. Young’s modulus was measured using atomic force microscopy (AFM) increased in the aging group (Figure 7A). The reaction force of cells tested using a colloid probe with a ball stuck pressed cells increased in the aging group compared with the normal one (Figure 7B). Phalloidin staining depicted that the cytoskeleton became disordered and underwent disorganized expansion in the aging group compared with the normal group *in vivo* and *in vitro* (Figure 7C, 7D). The immunofluorescence images of fibrous actin (F-actin), an important component of the cytoskeleton, showed increased levels in aging models compared with normal cells or tissues (Figure 7E, 7F). Western blot revealed that F-actin expression increased in the aging group compared with normal one *in vivo* and *in vitro* (Figure 7G). Yes-associated protein (YAP) and transcriptional co-activator with PDZ-binding motif (TAZ) are sensors of the mechanical forces. They can act as force-sensitive anchors or transporters. Western blot demonstrated that YAP and TAZ expression increased in the aging group compared with the normal one *in vivo* and *in vitro* (Figure 7H). circANKRD42 accelerates pulmonary fibrosis via YAP/TAZ-mediated mechanical stiffness [14], so we tested its expression in the aging cell model. qRT-PCR results revealed that circANKRD42 expression also increased in the aging cell model compared with the normal one (Figure 7I). These data demonstrated that aging induced cytoskeleton reorganization and increased the mechanical forces *in vivo* and *in vitro* through the circANKRD42-mediated YAP/TAZ signal pathway. Finally, the change in circANKRD42 expression was further measured in a healthy-population group. Results showed that circANKRD42 expression increased with age (Figure 8A). Meanwhile, cytoskeleton and F-actin were observed under a laser confocal microscope. The cytoskeleton became disordered and underwent disorganized expansion and F-actin increased in 60-year-old healthy people compared with 11-year-old healthy ones (Figure 8B, 8C). The above data suggested that circANKRD42 also mediated the mechanical forces in aging the same as in pulmonary fibrosis.

## DISCUSSION

Age is the most important risk factor for pulmonary fibrosis [20–22]. However, the fibrotic molecular pathways in aging have not been researched in depth. Thus, investigating the fibrotic players underlying aging is necessary. In the present study, based on the screened differentially expressed proteins by mass spectrometry, we demonstrated that the expression of aging genes and fibrotic genes were gradually upregulated during senescence. We further explored the fibrotic molecular pathways related to ferroptosis, autophagy, mitochondrial, and mechanical forces in aging based on our previous research about pulmonary fibrosis (Figure 8D). Our findings elucidated that aging can trigger changes in pulmonary fibrotic genes and contribute to the onset and progression of pulmonary fibrosis.

### Mechanical forces in aging

In correlation with body aging, lung tissue gradually ages and undergoes a series of changes in structure and function. For example, lung hardness increases gradually but elasticity and FVC decrease [23, 24]. These changes are also significant characteristics of pulmonary fibrosis [25, 26]. Throughout pulmonary-fibrosis progression, lung tissue becomes rigid from the lung periphery and progresses toward the lung center. Stiffening is caused by cellular mechanical stress such as cellular-matrix accumulation, cytoskeleton rearrangement, matrix hardness, compressive stress, and so on, thereby inducing a change in lung elasticity [27, 28]. Wu et al. demonstrated that elevated mechanical tension activates a TGFβ1 signaling loop in alveolar type II cells, driving the periphery-to-center progression of lung fibrosis [29]. In fact, cells of the human body experience mechanical forces throughout their lifespan, and these forces prevail in the cell microenvironment [30, 31]. In our previous study [14], changes in mechanical stiffness have been measured through Young’s modulus by using an atomic force microscope. Quantitative measurements proved that the increase in cell stiffness is accompanied by cytoskeleton rearrangement in TGFβ1-induced pulmonary fibrosis. In the current work, *in vitro* and *in vivo* experimental results also demonstrated that mechanical stiffness increased and the cytoskeleton became disordered and disorganized during aging. We further explored senescent human lung tissues, just as with the results of *in vitro* and *in vivo*.

Numerous studies report that chemical and mechanical signals can activate cell ultrastructures such as cytoskeleton, autophagosome, and mitochondrion, thereby driving the cell biological processes of cell proliferation, migration, and death [32–34]. Thus, mechanotransduction entails the conversion of mechanical cues into specific biochemical cues [35]. Liu et al. demonstrated that the mechanical cue shear stress can elicit vascular endothelial cell autophagy via redox regulation and Sirtuin-1 expression [36]. Adhesion-mediated mechanosignaling forces activate a mitochondrial stress response via solute carrier family 9 member A1-dependent ion exchange and heat shock factor 1-dependent transcription [37]. The abnormalities of cell ultrastructure can alter biological processes. For example, FUN14 domain containing 1 promotes hepatocyte injury through GPX4 binding to facilitate its mitochondrial translocation through the translocase of the outer membrane/translocase of the inner membrane complex, where GPX4 is degraded by mitophagy to trigger ferroptosis [38]. The present study revealed that aging induced an increase in mechanical force and ferroptosis, autophagy blockade, and mitochondrial dysfunction. These abnormalities often appear in the occurrence and development of pulmonary fibrosis.

### Epigenetic regulation in aging

Notably, epigenetics studies have grown exponentially over the last decade. The most recognized epigenetic regulations are DNA methylation, histone modifications, noncoding RNAs (ncRNAs), and chromatin remodeling. Acquired or inherited epigenetic changes can regulate gene expression without modifying the DNA sequence itself; they can be long lasting, but they can also be potentially reversible. Loss of epigenetic information is considered as a hallmark of aging [1, 39]. Yang et al. developed systems to reset epigenetic information in cells and mice. Epigenetic reprogramming can restore a youthful epigenome in ICE mice leading to reverse aging [40]. The current work revealed that ncRNAs such as increased lncIAPF and circANKRD42 underwent changes in themselves and regulated the changes in biological processes. Future research may be devoted to the precise manipulation of the epigenome to reverse aging and thus treat or cure age-related diseases. Our studies of abnormalities in mechanical force and ferroptosis, autophagy blockade, and mitochondrial dysfunction will help to enhance our understanding of aging-induced pulmonary fibrosis, thereby guiding the use of anti-aging as an early intervention for pulmonary fibrosis and age-related diseases.

### Clinical implications

In summary, we identified the key molecular players and signaling pathways such as epigenetic alteration, mitochondrial dysfunction, autophagy blockade, and increased ferroptosis and mechanical forces, which were involved in aging and pulmonary fibrosis. Our work depicted a comprehensive cellular and molecular atlas of the aging lung and provided insights into senescence-inducing pulmonary fibrosis. However, the limitation is that no in-depth study has been conducted on which and how aging phenotype is more likely to cause pulmonary fibrosis. In the future, we will continue to improve our research to attempt at elucidating which and how aging phenotype easily triggers uncontrollable pulmonary fibrosis and thus provide reference for research on the elderly susceptible to pulmonary fibrosis.

## MATERIALS AND METHODS

### Animal model and ethics statement

All C57BL/6 male mice were purchased from Jinan Tengli (Jinan, China), with 10 mice in each group. Among them, 2-month-old mice and 16-month-old mice were directly used for experiments. Pulmonary fibrosis mice were sprayed with 5 mg/kg BLM into the lungs using Penn-Century MicroSprayer (Penn-Century Inc., Wyndmoor, PA, USA), On the 28th day of modeling, the lung changes of all mice were evaluated by a MicroCT imaging system for small animals (PerkinElmer, USA). Then, mice were anesthetized by intraperitoneal injection of 2.5% avertin (0.25 g/kg). Lung samples were collected, the left lungs were fixed in 4% paraformaldehyde for histopathological sectioning, and the right lungs were stored at −80°C for protein extraction. Animal experiments were approved by the Animal Experimentation Ethics Committee of the Binzhou Medical University Hospital (No. 20200128-19).

### Patient samples

Blood samples and paraffin sections of human lung tissues were obtained from the Binzhou Medical University Hospital. Blood samples were collected from healthy individuals aged 20-40, 40 to 60, and 60-80 years; paraffin sections were collected from healthy individuals aged 11 and 60 years. This part of research was approved by the Ethics Committee of Binzhou Medical University (NO. 2021-235).

### H&E and Masson’s trichrome staining

The isolated tissue blocks were fixed in 4% paraformaldehyde as soon as possible for approximately 2 hours and then placed in a refrigerator to continue fixation for 48 hours at 4°C. The fixed mice lung tissues were dehydrated and soaked in paraffin overnight. The following day, lung tissues were cut into 4 μM sections using a Leica (RM2255) slicer and stained with H&E or Masson trichrome staining kits (Solarbio, China), respectively. The stained sections were dehydrated with anhydrous ethanol, cleared in xylene, dropped with neutral gel, and sealed with coverslips. The lung tissues of each group were observed under light microscopy.

### FVC testing

After the mouse underwent intraperitoneal anesthesia, a small incision was made in the trachea of the mouse, and a metal cannula was inserted into the small incision. Then, a plastic catheter was connected to a lung maneuver (DSI Buxco, USA). Mechanical ventilation was performed with a breathing rate of 150 breaths/min, 10 mL/kg tidal volume, and 3 cm H_2_O PEEP. The negative pressure-driven forced expiratory (NPFE) maneuver was applied. Mouse lung was inflated to a pressure of 30 cm H_2_O for 2 s, then the airway was connected to the negative pressure reservoir (−50 cm H_2_O) for 2 s. FVC was calculated directly from the flow cycle generated during lung deflation.

### MicroCT measurement

The MicroCT imaging system for small animals (PerkinElmer, USA) was warm-up and the parameters were adjusted in advance. After the mice were subjected to intraperitoneal anesthesia, they were positioned flat on the MicroCT machine. The X-ray parameters were adjusted to 90 KV and 88 μA. The resolution of CT images was 36 mm FOV, with an exposure time of 4 min. Two-dimensional tomographic images were obtained by using imaging software (SimpleViewer). Three-dimensional reconstruction was performed by using Analyze 11.0 (AnalyzeDirect, USA). Finally, the MicroCT images were taken and outputted.

### Immunofluorescence observation

MRC-5 cells were inoculated into 24-well plates, and paraffin sections of mouse or human lung tissues were baked for dewaxing, fixed with 4% paraformaldehyde fixative, punched with TritonX-100 and then washed 2–3 times with PBS, and then closed with goat serum for 1 h. Primary antibodies were added, and the plates were incubated at 4°C overnight with antibodies: anti-α-SMA (1:200, Affinity, AF1032), anti-S100A4 (1:250, Affinity, DF6515), anti-F-actin (1:250, Abcam, ab130935), anti-SPC (1:250, Affinity, DF6647), anti-E-cadherin (1:250, Affinity, BF0219), P16 (1:250, Affinity, BF0580). On the next day, fluorescently labeled secondary antibody was added and incubated for 60 minutes at room temperature protected from light. Cell nuclei were stained by adding DAPI and then washed with 1 × PBS. Finally, a drop of anti-fluorescence quencher was applied to the cell slides. All images were collected under fluorescence microscopy (Invitrogen EVOS M7000, Thermo Fisher, USA).

### Liquid chromatography-mass spectrometry (LC-MS) analysis

Proteomic analysis was performed by the Jingjie PTM BioLab Co., Ltd., (Hangzhou, China). Fresh lung tissue samples from 2-month-old and 16-month-old mice were used for the protein preparation. The proteins from lung tissue were quantified by using a BCA assay kit (Biyuntian, China). Protein samples were reduced with 5 mmol/L dithiothreitol for 30 min at 56°C, then stewing 15 min with 11 mmol/L alkylated iodoacetamide. The protein solution was then digested into trypsin. Peptides were solubilized with liquid chromatography mobile phase A and then separated using an EASY-nLC 1200 ultra-high performance liquid phase system. Phase A consisted of an aqueous solution of 0.1% formic acid and 2% acetonitrile, while phase B consisted of an aqueous solution of 0.1% formic acid and 100% acetonitrile. The liquid phase gradient was set as follows: 0–68 min, 6–23% B; 68–82 min, 23–32% B; 82–86 min, 32–80% B; 86–90 min, 80% B; and the flow rate was maintained at 500 nL/min. The data acquisition mode was performed using a cycle time-based data-dependent scanning procedure, i.e., the peptide parent ions were selected in order of signal intensity from highest to lowest in a 1.0 s cycle period to be sequentially fragmented into the HCD collision cell using a fragmentation energy of 27%, again sequentially for secondary mass spectrometry.

LC-MS/MS data processing was handled using the Proteome Discoverer search engine (v2.4.0.305). Tandem mass spectra were searched against the Mus musculus database (17,063 entries) and the reverse decoy database. Proteomics annotation and functional enrichment analysis was performed using the UniProt GOA database (http://www.ebi.ac.uk/GOA/) and InterProScan software (http://www.ebi.ac.uk/interpro/). Functional descriptions of the identified protein structural domains were annotated by InterProScan software based on the InterPro structural domain database. Subcellular localizations, such as cytoplasm, nucleus, mitochondria, Golgi apparatus, and endoplasmic reticulum (ER), were predicted by the WoLF PSORT software (https://wolfpsort.hgc.jp/). The Kyoto Encyclopedia of Genes and Genomes (KEGG) database (https://www.kegg.jp/) was further used to annotate protein pathways. In pairwise comparisons, proteins with a fold change greater than 1.5 and an adjusted *p*-value < 0.05 were defined as differentially expressed proteins.

### MRC-5 cell culture and treatment

The human fetal lung fibroblast MRC-5 cell line was obtained from the America Typical Culture Collection. 5–10 generations of cells were selected for the experiment. The cell medium contained 10% fetal bovine serum, 1% gluta-max, 1% NEAA, 1% sodium pyruvate, and 100 × penicillin/streptomycin. The cells were cultured at 37°C in the 5% CO_2_ incubator and were divided into normal, TGFβ1-treated and etoposide groups. Cells in the TGFβ1-treated group were stimulated with 5 ng/mL TGFβ1 for 72 hours; cells in the etoposide group were treated with 10 μM etoposide (MedChemExpress, Shanghai, China) for 72 hours.

### Senescence-associated β-galactosidase detection

SA-β-Gal Staining Kit (catalog number G1580, Solarbio, Beijing, China) is a staining assay for senescent cells or tissues based on the up-regulation of SA-β-Gal activity levels during senescence, which uses X-Gal as a substrate and generates a dark blue product catalyzed by senescence-specific β-galactosidase. Aging cells were identified by the senescence-associated β-galactosidase kit. Cells were cultured in 6-well plates, fixed and stained using a senescence-associated β-galactosidase kit according to the manufacturer’s instructions. The images were captured under an inverted microscope (Germany Ika). Aging cells were identified and quantified by the positive SA-β-Gal staining cells. The displayed images were representative of 3 independent experiments.

### Western blot

Cells or lung tissues were collected and lysed in radioimmunoprecipitation assay (RIPA) buffer and phenylmethylsulfonyl fluoride (PMSF) (RIPA buffer: PMSF = 100:1). Protein concentrations were determined using the diquinolinic acid protein assay kit (Coolaber, China). After separation in sodium dodecyl sulfate-polyacrylamide gel electrophoresis, the proteins were transferred to PVDF after 120 min using a current of 200 mA. The proteins were sealed with 5% skim milk for 2 hours and incubated overnight at 4°C with anti-collagen III (1:1000, Affinity, China, AF5457), anti-collagen I (1:1000, Affinity, China, AF7001), anti-α-SMA (1:1000, Affinity, China, AF1032), anti-vimentin (1:1000, Affinity, China, AF7013), anti-GAPDH (1:10,000, Affinity, China, AF7021), Anti-SPC (1:1000, Affinity, China, DF6647), anti-FAP1 (1:1000, Cell Signaling, USA, 66562), anti-TGFβ1, anti-DRAM (1:1000, Cell Signaling, USA), anti-GABARAP (1:1000, Affinity, China, DF7419), anti-LC3A/B, anti-E-cadherin (1:1000, Affinity, China, BF0219), anti-PINK (1:1000, Affinity, China, DF7742), anti-C-Jun, anti-NRF1, anti-COX-IV (1:1000, Affinity, China, AF6090; AF5298; AF5468), anti-S100A4 (1:1000, Affinity, DF6515), anti-P16 (1:1000, Affinity, BF0580), anti-TGFβ1 (1:1000, Proteintech, China, 21989-1-AP), anti-SOD2 (1:1000, Proteintech, China, 24127-1-AP), anti-DRP1 (1:1000, Proteintech, China, 12957-1-AP), anti-TAZ (1:1000, Proteintech, China, 23306-1-AP), anti-OPA1 (1:1000, Proteintech, China, 27733-1-AP), anti-SMP30 (1:6000, Proteintech, China, 17947-1-AP), anti-ATF3 (1:500, Abcam, UK, ab254268), anti-F-actin (1:500, Abcam, UK, ab130935), anti-STAT1 (1:1000, Abcam, UK, ab234400), anti-MMP9, or anti-P62 (1:10000, Abcam, UK, ab109012). The membranes were washed with 1× Tris Buffered Saline Tween three times and incubated with secondary antibodies at room temperature for 1 h. The expression of the proteins was detected by an enhanced chemiluminescence reagent kit (Spark Jade, China).

### RNA binding protein immunoprecipitation (RIP)

RIP was performed under the guidance of the RNA immunoprecipitation kit (Genesee, Beijing, China). Cells were collected and lysed. The supernatant was aspirated after centrifugation at 12,000 rpm for 10 minutes. Protein A+G beads were pretreated and ligated with HuR and immunoglobulin G (IgG) antibodies for 3–4 hours respectively. Protein A+G beads conjugated with HuR and IgG antibodies were added to the cell lysate supernatant overnight. RNA was purified and extracted, then reverse transcribed using the Evo M-MLV Reverse Transcription Kit (Accurate, Changsha, China) and RT-PCR was performed to detect the expression levels of the target genes.

### Phalloidin staining

MRC-5 cells and lung tissue were fixed with 4% formaldehyde for half an hour, rinsed twice in PBS, and permeabilised with 0.1% Triton-X 100 (PBT, pH = 7.3) in PBS for half an hour. The appropriate amount of phalloidin staining solution was added to the cells and incubated for half an hour at room temperature. The cells and tissues were stained with DAPI and blocked by a drop of anti-fluorescent bursting agent on the cell slides. Images were observed under a laser confocal microscope (Zeiss LSM880, Germany).

### Mitochondrial membrane potential assay

Collect MRC-5 cells, centrifuge at 1000 rpm for 5 min and discard the supernatant, add culture medium to resuspend the precipitate. An equal volume of JC-1 staining solution was added, mixed well and incubated at 37°C for 20 min, centrifuged at 600 g at 4°C for 3 min, and the supernatant was discarded. Each sample was washed twice by adding 1 mL of 1× JC-1 staining buffer, centrifuged at 600 g for 3 min at 4°C, and the supernatant was discarded. The cells were resuspended by adding appropriate amount of JC-1 staining buffer. The fluorescence images were detected under a laser scanning confocal microscope (Zeiss LSM880, Germany).

### Mitochondrial reactive oxygen species (MitoROS) detection

MitoROS was detected by MitoSOX red reagent (ThermoFisher Scientific, USA, M36008). MRC-5 cells were inoculated into sterile six-well plates and cell senescence was induced with 10 μM etoposide when cell density was reached up to 80%. Cells were collected and incubated for 10 min with MitoSOX Red Reagent Probe Solution and analyzed by flow cytometry (Becton, Dickinson and Company, USA).

### Detection of intracellular iron concentration

Intracellular iron concentration was measured using Phen Green cells SK (PGSK, Cayman, USA). Cells were collected, washed twice with PBS, and stained with PGSK for 10 min in the dark before flow cytometry analysis (Becton, Dickinson and Company, USA).

### Measurement of total iron

About 0.1 g of lung tissues added with 0.9 mL of reagent IV was homogenized. The supernatant was aspirated after centrifugation at 12,000 g for 10 minutes according to the Total Iron Colorimetric Assay Kit (Elabscience Biotechnology Institute, China). The OD value was measured at 593 nm by using a microplate reader, the total iron content in lung tissues of normal or senescent mice was determined according to the test OD value.

### Young’s modulus measurement by AFM

MRC-5 cells were cultured at WillCo-dish glass bottom dishes (Willco Wells BV, GWST-5040). Cell images were captured in QI mode under an AFM (JPK NanoWizard 4, Bruker Nano GmbH, Germany). A PFQNM-LC-CAL probe (Bruker Nano GmbH) with an end radius of 75 nm and a force constant of 0.09 N/m was used to acquire topographical images. After an entire cell was imaged by AFM in PBS, the colloid probe (MLCT-O-A probe, Bruker Nano GmbH) with a 20-μm diameter silica sphere was localized on an intact single cell. Young’s modulus was measured using the AFM indentation test based on a Hertz model.

### Measurement of MDA

About 0.05 g lung tissues of 2-month-old mice and 16-month-old mice were lysed and homogenized with 500 μL of Beyotime’s Western lysis solution, and the supernatant was aspirated by centrifugation at 12,000 g for 10 min. The MDA content was determined by the Lipid Peroxidation MDA Assay Kit (Beyotime Biotechnology Institute, China) according to the manufacturer’s instructions.

### qRT-PCR analysis

Total RNA was extracted from MRC-5 cells using Trizol and reversed transcribed to cDNA using a reverse transcription kit (TaKaRa Biotechnology, Japan). The total volume of the 20 μL system was as follows: 2 μL cDNA, 7.2 μL RNA-free water, 0.4 μL lncIAFP/GAPDH forward primer, 0.4 μL lncIAPF/GAPDH reverse primer, 10 μL SYBR^®^Premix Ex Taq™. The reaction procedure was as follows: keep the temperature at 95°C for 30 seconds, perform 45 PCR amplification cycles at 95°C for 5 seconds, 60°C for 20 seconds and 72°C for 30 seconds. The primers for the lncIAPF are as follows: 5′-CTACCTCAAGCCTTACTTCCTCCG-3′, 5′-GAATACAAGGCGCTATGCTAGGAAC-3′. The primers for the circANKRD42 are as follows: 5′-GGAGAGACCCAGTGATGTGG-3′, 5′-TCATCCTGGGCTGTCAGATTTGCTC-3′.

### Statistical analysis

Data were expressed as the means ± standard deviation (SD) and analyzed using the SPSS 21.0 statistic software program. *T*-tests were used to assess overall differences between groups and make post hoc comparisons. *P* < 0.05 was considered statistically significant.

### Availability of data and materials

All data associated with this study are present in the paper.
